# The effect of cumulative energy in repeated subliminal transscleral cyclophotocoagulation: a retrospective study

**DOI:** 10.1186/s12886-024-03505-7

**Published:** 2024-06-03

**Authors:** Áron Szabó, Tamás Árpádffy-Lovas, Krisztina Hagyó, István Cseke, Péter Vámosi, Edit Tóth-Molnár

**Affiliations:** 1 Department of Ophthalmology, University of Szeged, Szeged, Hungary; 2 Department of Ophthalmology, Péterfy Hospital, Budapest, Hungary; 3 Department of Ophthalmology, Erzsébet Teaching Hospital, Sopron, Hungary

**Keywords:** Transscleral cyclophotocoagulation, Cumulative energy, Fluence, Silicone oil induced glaucoma

## Abstract

**Background:**

The long-term safety and efficacy of repeated applications of subliminal transscleral cyclophotocoagulation (SL-TSCPC) with a focus on cumulative energy was evaluated in glaucoma patients.

**Methods:**

In this retrospective, multicentric study the data of a total of 82 eyes with various causes of glaucoma that were treated with a single or multiple applications of SL-TSCPC were collected. Treatments were performed under general or local anesthesia with an 810 nm diode laser. Power was 2000 mW; duty cycle, 31.3%; total treatment duration, 80–320 s; equaling a total energy of 50–200 J per treatment session. Fifty-five eyes (55 patients) presented for all follow-ups, and these eyes were selected for further statistical analysis. The mean age was 60.0 ± 17.1 years, and 22 (40%) of the patients were female. Intraocular pressure (IOP) and dependence on further glaucoma medication were evaluated at 12 months following the initial treatment.

**Results:**

Eyes underwent 1 or 2 consecutive SL-TSCPC treatments. Median (min–max) baseline IOP of 34 (13–69) decreased to 21.5 (7–61), 22 (8–68), 20 (9–68), and 19.5 (3–60) mmHg at the 1, 3, 6, and 12-month postoperative timepoints respectively. The mean (± SD) IOP decrease at 12 months was 26 ± 27%, 39 ± 32%, and 49 ± 33% in the low (below 120 J, *n* = 18), medium (120–200 J, *n* = 24), and high (above 200 J, *n* = 13) cumulative energy groups respectively. At the 12-month timepoint, oral carbonic anhydrase use was discontinued in ¾ of the cases.

**Conclusions:**

It was found that the repeated application of SL-TSCPC safely and efficiently decreases IOP in a Caucasian population with heterogenous causes of glaucoma, eyes with silicone oil responded to a greater extent. Inclusion of cumulative energy scales may contribute to better addressing repeated procedures in a standardized fashion.

## Background

 Current glaucoma treatment modalities aim to reduce the intraocular pressure (IOP) [1–3] by increasing the outflow of the aqueous and/or decreasing its production. The classical therapeutic regimen usually consists of topical medication [4] or selective laser trabeculoplasty (SLT) [5] as first-line options. Second-line options should the conservative first-line approaches fail to reduce IOP to the target level [6], include oral carbonic anhydrase inhibitor (CAI) treatment [7] and invasive surgical techniques, such as trabeculectomy [8] and the implantation of drainage devices [9, 10]. Cyclodestructive procedures have been considered as a last step in controlling IOP, as these procedures are often associated with severe adverse events [11, 12]. With the advent of subliminal transscleral cyclophotocoagulation (SL-TSCPC), the incidence of complications appear to be lower compared to continuous wave transscleral cyclophotocoagulation (CW-TSCPC), making it a plausible choice for patients in whom other surgical options would be less feasible [13]. The efficacy and the safety of SL-TSCPC treatment in primary open-angle glaucoma (POAG) [14–16] seem to be favourable, and the results in secondary glaucoma cases have also been promising [17, 18]. The delivered treatment energy shows large variations in power, duration, and duty cycle [19–23]. Sweep velocity and the resulting fluence are also paramount factors to be considered, as higher fluence is associated with greater efficacy [24]. According to the hypothetical search for ideal parameters by Sanchez et al., the target energy should be approximately 150 J [25]. While Lim et al., who have investigated repeated SL-TSCPC procedures and have introduced the concept of cumulative energy, postulated higher values of 150–200 J [26].

Expert consensus guidelines were published [24, 27] after the examined treatment period and during the data processing period of this study, and these guidelines recommend a total energy of 125.2 J. Alongside the importance of fluence, we would also advocate that cumulative energy, as proposed by Lim, should be included when designing future studies regarding the safety and efficacy of SL-TSCPC treatment.

In this work, we investigated the clinical response to SL-TSCPC treatment in various types of IOP rise.

## Methods

### Data collection

A retrospective, multicentric analysis of 82 eyes of 82 Caucasian patients undergoing SL-TSCPC between 2019 and 2021 at three regional ophthalmology centers (University of Szeged, Szeged, Hungary, Péterfy Hospital, Budapest, Hungary, and Erzsébet Teaching Hospital, Sopron, Hungary) was performed (Table 1). The Ethics Committee of the University of Szeged approved the data acquisition with the participation of the ancillary sites (protocol number: SLTSCPCRET-001; file number: 34/2022SZTE RKEB). The study was conducted in accordance with the Tenets of the Declaration of Helsinki. Baseline characteristics, including the type of glaucoma (Table 2), the age and sex of the patient, as well as visual acuity (VA), IOP, topical and oral antiglaucoma medication were noted. Furthermore, the following interventional parameters were collected: anesthesia, laser machine used, marking method of the treatment area, energy, duty cycle, duration, postoperative care regimen. Follow-up visit times were defined at postoperative months 1, 3, 6 and 12. At each follow-up, VA, IOP, topical and oral antiglaucoma medication and adverse event data were registered for further analysis.

**Table 1:** Demographic information

	No. eyes (no. patients)	82	(82)
**Completed all follow-ups over 12 months**:			
	No. eyes over (no. patients)	55	(55)
	Mean age (SD) (y)	60	(17)
	Median (range) (y)	61	(7–88)
	Female sex [n (%)]	22	(40%)

**Table 2:** Distribution of Glaucoma Types

Glaucoma diagnosis	*n*	(%)
Silicone oil induced	31	(38)
Neovascular	16	(20)
Primary open-angle	14	(17)
Uveitic / Inflammatory	6	(7)
Primary closed-angle	4	(5)
Congenital	3	(4)
Other secondary glaucoma	2	(2)
Pigment dispersion	2	(2)
Traumatic	2	(2)
Aphakic glaucoma	1	(1)
Pseudoexfoliation	1	(1)

Criteria for success were defined as 20% or greater reduction in IOP (criterion A), 25% or greater reduction in IOP (criterion B), and 30% or greater reduction in IOP from baseline (criterion C), with or without topical glaucoma medications at follow-up as published by Tekeli et al. [19] Target IOP values at or under 18, 15, and 12 mmHg were defined as a secondary outcome measure, also based on the same publication [19].

Complete failure was defined as IOP lower than 6 mmHg with hypotony maculopathy (criterion 1), loss of three or more Snellen lines or loss of light perception (criterion 2), or surgical failure when additional glaucoma surgical intervention to control the IOP was needed, or discontinuation of oral CAI was not possible (criterion 3), as published by Jun Yong Chow et al. [28] and modified to this study so that criterion 3 excluded repeated SL-TSCPC or other non-invasive lasers, such as SLT.

### SL-TSCPC procedures

Treatments were performed under general or local (retrobulbar, peribulbar or sub-Tenon’s block) anesthesia with either the Supra 810 or the Vitra 810 systems (*n* = 77) and the Subcyclo probe (Quantel Medical, Rockwall, TX, USA) or the Cyclo G6 Laser System (*n* = 5) with the original MicroPulse P3 probe (IRIDEX Corporation, Mountain View, CA). Power and duty cycle were constant at 2000 mW and 31.3%, respectively. The treatment area was defined with the aid of transillumination; eyes not suitable for visualizing the ciliary body band were marked 3 millimeters posterior to the limbus, with 3 and 9 clock hours omitted in all cases. Total treatment duration ranged from 80 to 320 s, equaling a total energy of 50 to 200 J per treatment session, delivered via a slow continuous sweeping motion.

Two subgroups were formed based on the number of total SL-TSCPC sessions: the first subgroup participated in a single session, the other subgroup consisted of eyes that received one additional treatment during the follow-up period. Cumulative energy was calculated in eyes undergoing repeated treatment sessions. The follow-up time commenced from the first session of the study period.

### Statistical analysis

IOP data is expressed as median (minimum–maximum), and other data is expressed as mean ± standard deviation (SD). The homoscedasticity of each variable was confirmed using Bartlett’s test, and the normality of their distribution was confirmed using the Shapiro-Wilk test. Statistical analysis was performed in R (Vienna, Austria), using RStudio (PBC, Boston). Cumulative success and failure in all 82 eyes were evaluated using Kaplan–Meier survival analysis and log-rank test. Further statistical analysis was performed on the 55 eyes that completed the 12-month follow-up. Changes in IOP throughout the follow-up period were treated as repeated measurements and were analysed using ANOVA followed by Bonferroni-corrected t-tests. When calculating the required IOP decrease (%) to fulfil criteria, the required percentages were calculated for each patient, and these percentages are presented as mean ± SD. The decrease in oral CAI use and subgroup analyses for silicone oil eyes were evaluated using Fisher’s exact test. Other possible explanatory variables were also analysed using Fisher’s exact test and logistic regression analysis. Statistical significance was assumed when the p-value was < 0.05.

## Results

A total number of 104 SL-TSCPC treatments on 82 eyes were reviewed: 82, 76, 66 and 55 eyes had 1, 3, 6 and 12 months of follow-up respectively.

**Fig. 1:**
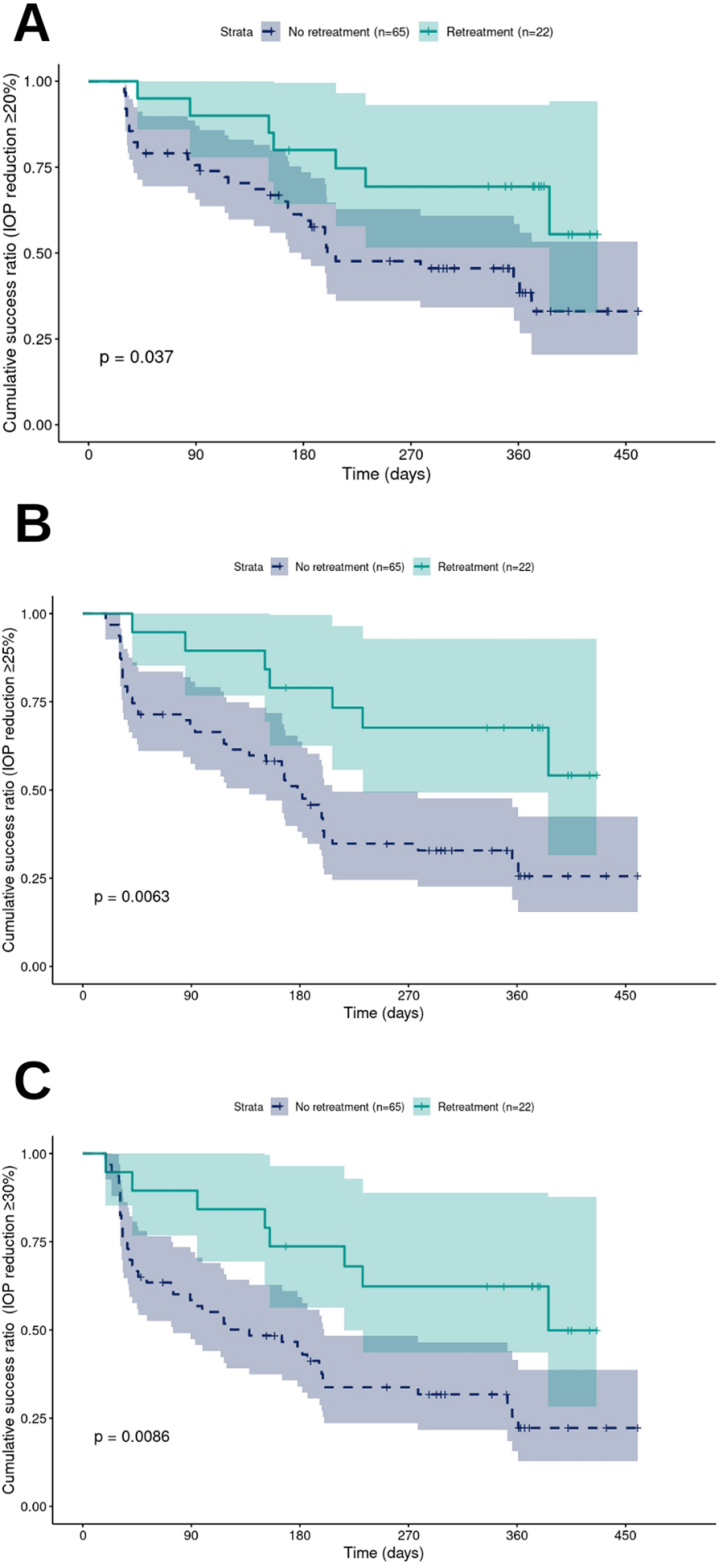
Kaplan–Meier survival analysis based on success criterion A, 20% or greater reduction in IOP (**A**); criterion B, 25% or greater reduction in IOP (**B**); and criterion C, 30% or greater reduction in IOP (**C**); and failure criteria 1-3; p-values calculated using log-rank test

Kaplan–Meier survival analysis performed on 82 eyed showed at least 25% success in all eyes for all success criteria. Repeated treatment showed statistically significant additional benefit (Fig. 1).

A substantial portion of the 82 eyes presented with secondary glaucoma induced by silicone oil (*n* = 31) A variety of other etiologies accounted for the remaining cases (Table 3).

**Table 3:** Prior History of Ocular Procedures

		*n*	(%)
Prior glaucoma surgery			
	Prior trabeculectomy	13	(12)
	Prior SLT	11	(13)
	Prior cyclophotocoagulation	3	(4)
	Prior drainage implant	3	(4)
	Prior LPI	3	(4)
	Prior cyclodestruction	2	(2)
	Prior combined cataract surgery and trabeculectomy	1	(1)
Prior cataract surgery		64	(78)
Prior PPV		37	(45)
Prior PPV + silicone oil implantation		31	(38)
Prior PRP		10	(12)
Prior intravitreal injection		9	(11)
Prior PRK		1	(1)
Prior DMEK		1	(1)
Prior trauma		1	(1)
Prior ECCE		1	(1)
Prior scleral buckling		2	(2)
Prior secondary artificial lens implantation		1	(1)
Treatment naive		6	(7)

*LPI* Indicates laser peripheral iridotomy, *PPV *Pars plana vitrectomy, *PRP *Panretinal photocoagulation, *PRK *Photorefractive keratectomy, *DMEK *Descemet's membrane endothelial keratoplasty, *ECCE* Extracapsular cataract extraction

Mean age at treatment was 60.0 ± 17.1 years. The male to female ratio was 22:33, and the right to left eye distribution was 26:29.

Of the 55 eyes that completed the 12-month follow up 37 (67%) underwent one, while 18 (33%) received two consecutive SL-TSCPC treatments depending on the clinical response and on the amount of IOP decrease. Thus, cumulative energy delivered throughout the sessions was calculated for each eye and the following thresholds were defined: under 120 J (mean delivered energy: 96.5 ± 10.9 J), 120–200 J (mean delivered energy: 186.4 ± 25.4 J) and above 200 J (mean delivered energy: 291.8 ± 65.3 J) as low (*n* = 18, 33%), medium (*n* = 24, 44%) and high (*n* = 13, 23%) cumulative energy groups respectively.

The postoperative treatment regimen consisted of dexamethasone, NSAIDs, cyclopentolate, and atropine in various combinations. Only 1 of the 55 eyes did not receive anti-inflammatory therapy.

VA was recorded on Kettesy’s chart, which is a decimal chart similar to Snellen’s ranging from 0.1 to 1.0 values, read from 5 m. Visual acuities below this were converted as follows; 0.08, 0.06, 0.04, 0.02 for counting fingers from 4, 3, 2 and 1 m respectively. Hand movement and light perception were given 0.001 and 0.0001 values respectively. No light perception equaled 0.

Median baseline IOP (34 (13–69) mmHg) decreased statistically significantly at 1 month (21.5 (7–61) mmHg) postoperatively and maintained a significant decrease through the 3-month (22 (8–68) mmHg) and the 6-month timepoints (20 (9–68) mmHg), all the way to the 12-month endpoint (19.5 (3, 4, 5, 60) mmHg; *p* < 0.001).

**Fig. 2:**
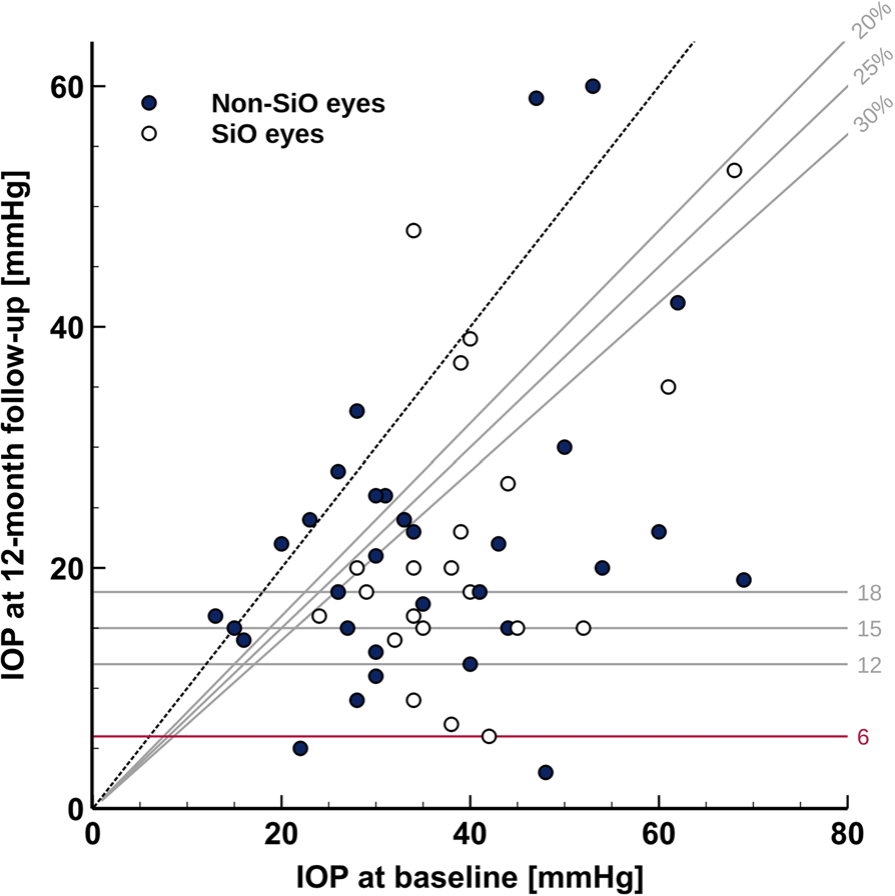
Scatterplot of pre-and postoperative IOP values; dashed line represents the limit below which IOP decrease was achieved, full diagonal lines represent 20%, 25%, and 30% decrease respectively, horizontal lines represent postoperative IOP values of 18, 15, 12, and 6 mmHg respectively

Success based on criteria A, B and C, with no failure criteria fulfilled, at 12 months were met in 27 (49%), 25 (45%), and 24 (44%) out of 55 eyes respectively. When secondary success endpoints were evaluated, eyes under or at 18 mmHg, under or at 15 mmHg and under or at 12 mmHg were 14 (25%), 8 (15%), and 6 (11%) respectively out of 55. (Fig. 2) Neither cumulative energy group did significantly outperform any other at each decrease threshold.

**Fig. 3:**
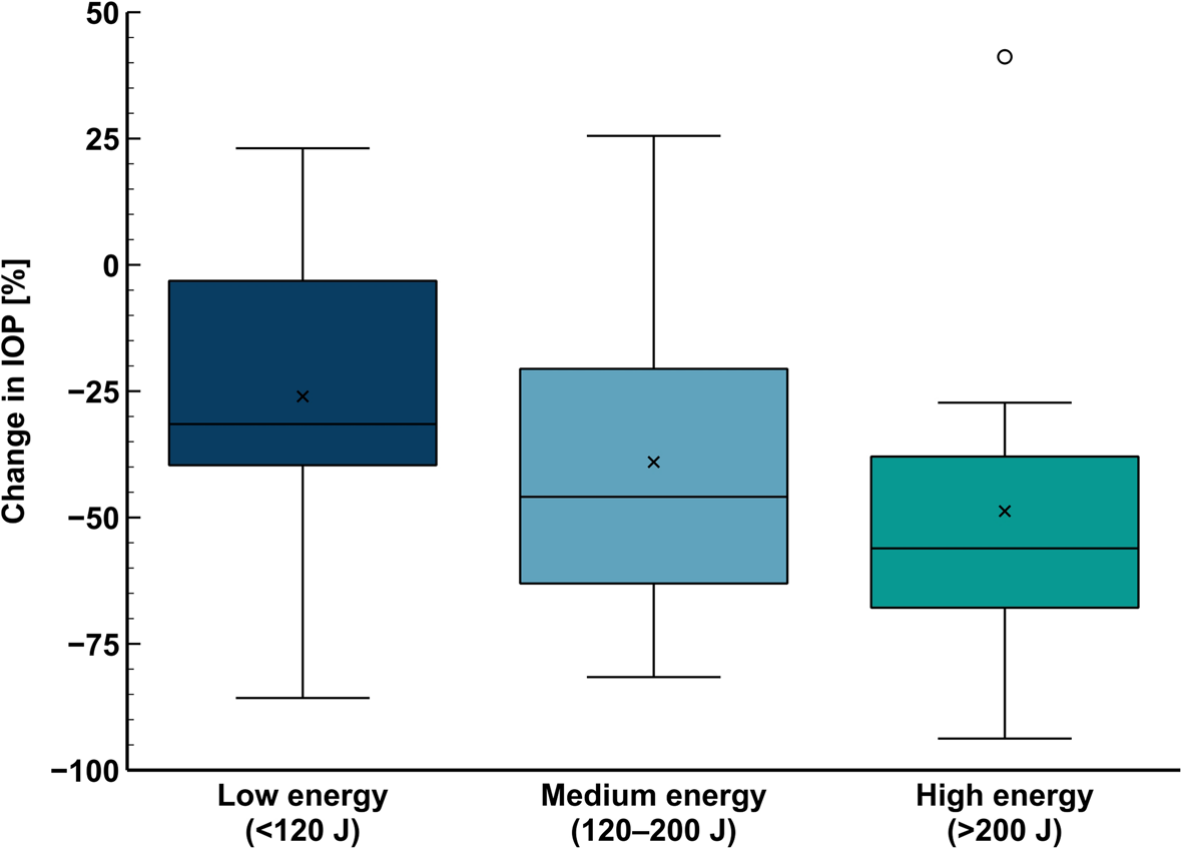
Changes in IOP at 12 months in the low, medium, and high cumulative energy groups

The mean decrease of IOP at 12 months was 26 ± 28%, 39 ± 32%, and 49 ± 33% in the low, medium and high cumulative energy groups respectively, with no statistically significant difference between the groups (ANOVA *p* = 0.17; Fig. 3). Median IOP at 12 months in the corresponding energy groups was 22 (6–42), 19 (5–60), 18 (3–48).

One eye (2%) with hypotony maculopathy (criterion 1), 17 eyes (31%) with a marked decrease in VA (criterion 2), and 6 eyes (11%) with surgical failure (criterion 3) were identified as treatment failures at 12 months. There was no statistically significant difference in the occurrence of failures between cumulative energy groups.

VA improved in 9 eyes (16%), decreased by 3 or more lines in 17 eyes (31%), and remained relatively stable in 29 eyes (53%). The magnitude of IOP decrease at 12 months was not correlated with VA loss of 3 or more lines decimal (failure criterion 2). Progression to no light perception (NLP) occurred in 5 patients during the 12-month follow-up. Preoperative VA was of hand motion or light perception in 4 of the 5 patients and 1 patient had 0.6 decimal vision that was lost after retinal detachment. Possible causes of decrease in VA, including baseline VA, baseline IOP, decrease in IOP, number of treatments, and cumulative energy were also evaluated using logistic regression. None of these variables were in interaction with the decrease in VA (Table 4).

**Table 4:** Changes in VA at 12 months after the first treatment

	Occurrence	%	Cumulative (%)
Lost to NLP	5	9.1	9.1
Lost to LP	2	3.6	12.7
-10 or worse	1	1.8	14.5
-6	2	3.6	18.1
-5	1	1.8	19.9
-4	2	3.6	23.5
-3	4	7.3	30.8
-2	1	1.8	32.6
-1	8	14.5	47.1
0	20	36.4	83.5
1 or better	9	16.4	100.0

Values < 0 number of lines of vision lost, Values > 0 number of line of vision gained. *LP* Indicates light perception, *NLP* No light perception

In the studied cohort, amaurosis fugax occurred in 1 patient, 3 patients presented with IOP under 6 mmHg after the procedure, and 2 of them retained preoperative VA with no maculopathy. Cystoid macular edema developed in 1 patient, age-related macular degeneration developed in 1 patient, diabetic retinopathy developed in 1 patient, central retinal vein occlusion (CRVO) developed in 1 patient, retinal detachment was seen in 1 patient, uveitis developed in 2 patients, and Meibomian gland dysfunction occurred in 1 patient.

**Fig. 4:**
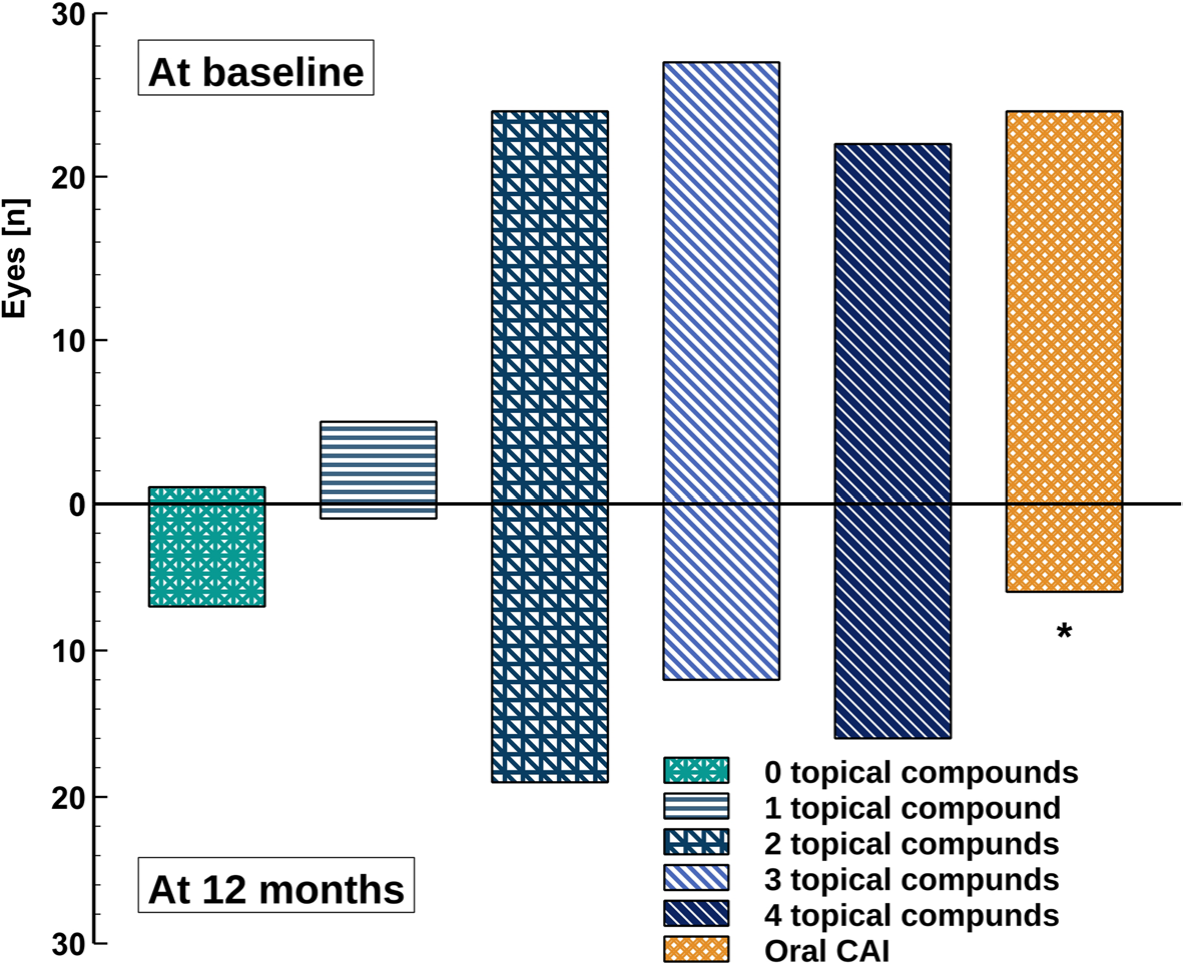
Changes in glaucoma medications from baseline to postoperative 12 months (CAI, carbonic anhydrase inhibitor); * – *p* < 0.05, Fisher’s exact test

There was a slight but not significant change recorded in the number of topical IOP lowering medications used at baseline and postoperative 12 months both in the number of bottles (1.7 ± 0.7 to 1.5 ± 0.5) and in the number of active ingredients (2.9 ± 1.0 to 2.5 ± 1.3). On the other hand, oral CAI use at postoperative 12 months had been successfully discontinued in 18 of the 24 patients previously in need of acetazolamide therapy (75% decrease, *p* < 0.01; Fig. 4).

The IOP decrease at 12 months in eyes with silicone oil (–43 ± 29%) was more pronounced compared to the rest of eyes (–33 ± 33%; *p* = 0.21). Criterion A success was also more common in eyes with silicone oil (Table 5, *p* = 0.03). These eyes had an odds ratio of 3.65 to reach 20% of IOP reduction compared to eyes with no silicone oil. No statistically significant difference was found between eyes with and without silicone oil regarding retreatments (*p* = 0.46) or the incidence of VA decrease (*p* = 1.00). Success in eyes with silicone oil was achieved with seemingly lower energy levels (162.8 ± 77.4 J) compared to non-silicone oil eyes (181.1 ± 58.9 J; *p* = 0.52).

**Table 5:** Criterion A success (–20% IOP) in eyes with and without silicone oil

	Eyes with silicone oil	Eyes without silicone oil	
Success	15	12	27
No success	7	21	28
	22	33	55

The following categoric variables were further evaluated using Fischer’s exact test: previous cataract surgery, prior glaucoma surgery, baseline oral CAI use, and sex; none of the variables were found to be associated with higher criterion A success (*p* = 0.36, *p* = 1.00, *p* = 0.42, and 1.00, respectively). Logistic regression was used to quantify the relative effect of the following continuous variables: cumulative energy, number of topical compounds at baseline, baseline IOP, baseline VA, and patient age. None of the variables were found to be associated with higher criterion A success (*p* = 0.33, *p* = 0.27, *p* = 0.64, *p* = 0.32, and *p* = 0.68).

No statistically significant difference was found between the two groups based on surgical treatment area, 3 mm posterior to the limbus (*n* = 34, 62%) versus transillumination (*n* = 21, 38%), in terms of mean IOP decrease, and fulfilment of success or failure criteria.

## Discussion

In this heterogeneous cohort of mainly secondary glaucoma patients with a high baseline IOP uncontrollable by prior therapy, the SL-TSCPC procedure offered a non-invasive, relatively safe method for an additional 26–49% decrease of IOP that could be observed from the first postoperative month all the way to the endpoint at 12 months. This effect is approximately equivalent to adding an extra topical medication or a fixed combination to the treatment regimen. While the number of glaucoma drops used could not be decreased significantly, the procedure enabled a large proportion of the patients to discontinue oral IOP lowering medication.

Despite the favourable success in achieving up to 30% of IOP reduction, the target IOP values of 18, 15, and 12 mmHg defined as secondary outcome measures were met in a smaller number of eyes. However, this may be attributable to the relatively high baseline IOP values, as in order to completely fulfil these outcome measures, the required IOP decrease was calculated to be −45 ± 22%,

–54 ± 18% and −63 ± 15% on average in all energy groups.

Similarly to the findings of Zbiba [17], the subgroup analysis of silicone oil induced IOP elevations showed decrease of more than 43%, even though silicone oil removal was not performed prior to or during the procedure. No underlying correlation was uncovered that can be added to the current existing hypothesis that the absence of vitreous gel promotes the posterior movement of the ciliary body during the laser procedure [17].

Failure seems to be independent of the cumulative energy within the thresholds defined in this study. Prolonged hypotony with additional complications, such as maculopathy, following SL-TSCPC is typically not encountered [28–30] or is resolved without sequale [14, 19] but should still be considered a valid risk (2–7%) as presented in this and other studies [21, 26]. Deterioration of VA (failure criterion 2) was observed in one third of patients. Large variation across publications exists from no VA loss [20] or low incidence [30] up to similarly high failure ratios [23, 26] Whether this can be solemnly attributed to disease progression, treatment related or non-related adverse events, or the combination of the former is not yet evident. The retrospective nature of our study did not allow for further differentiation between causes in this patient cohort with already severely compromised visual functions. Neovascular glaucoma and silicone oil induced glaucoma already pose a higher risk of vison loss [31, 32].

Review of the current literature shows that the criteria of success varies on a broad scale from IOP reduction of at least 20% [17, 18, 20, 23, 26, 28, 29, 33–39], IOP reduction of at least 30% [40–43], the variations of specific IOP and percentage limits [13, 19, 21, 25, 44], all the way to the most stringent criteria by Tekeli and Soussi [30, 45]. In our study, the fulfilment of these criteria shows the discrepancy between the high proportion of patients with an IOP reduction of more than 30% (65%) versus the percentage of low target IOPs achieved (13%) with this method alone. The large difference between Tekeli’s success ratio for their original criteria A, B and C of 67%, 53%, and 42% respectively compared to the equivalent of our combined primary and secondary endpoints of 35%, 18%, and 13% might be attributable to varying glaucoma subtypes, such as POAG vs. secondary glaucoma, and pre-treatment IOPs. Our results better resemble those of Soussi with 35%, 27%, and 11% despite dissimilar baseline characteristics. It should also be noted that both studies and our current cohort are in the fluence range [24] of ≤ 52.4 J/cm^2^, which might limit the overall IOP decreasing potential as well, compared to studies reporting SL-TSCPC efficacy with higher fluence values.

The preferred method of the authors was delivering 150 J of energy per session to the treatment area marked with transillumination with the power set at 2.0 Watts, the duty cycle at 31.3%, and treatment time at 120 s per hemisphere with a sweep velocity/hemisphere of 20 s/sweep, with 3 and 9 o’clock hours omitted. Repeated procedure was warranted if a minimum of 20% decrease was not seen or target IOP was not reached. Expert consensus guidelines published during the data acquisition and processing of this study recommended power of 2.5 Watts, duty cycle of 31.3% and treatment time of 80 s per hemisphere with a sweep velocity/hemisphere of 20 s/sweep [24, 27]. The calculated total energy, dwell times, and fluence of this study are 150 J, 0.63 s, and 51.89 J/cm^2^. The recommendation in the guideline for the same variables is 125.2 J, 0.63 s, and 64.86 J/cm^2^ respectively.

Fluence is greatly determined by surgical technique. Even in the presence of fixed power and duty cycle settings, substantial variability in delivered energy can arise from a number of confounding factors including surgeon experience, patient anatomy, identification of treatment area, aiming, real-time sweep velocity. Although aided by the periodic beeping of the SL-TSCPC machine or any other chronometer, actual dwell times are subject to unaccounted deviations from predefined protocol.

High-quality randomised clinical trials aim at recruiting patients with well-outlined inclusion and exclusion criteria, homogeneous glaucoma types, preferably no prior (or one specific) surgical procedures to ensure clarity of data. Our mixed retrospective cohort rather illustrates a real-life scenario, where multiple comorbidities, treatments, and large variations in baseline characteristics introduce uncertainty in the identification of explanatory variables.

Still, limited data are available on the relationship between the cumulative energy, the safety, and the efficacy of repeated procedures. A number of studies include a variable number of repeated SL-TSCPC treatments and cumulative energy values ranging from as low as 56.31 J [20] to as high as 800 J [21] (Table 6). The highest reported number of repeated interventions was four. One study by Lim [26] specifically focuses on the cumulative energy with up to three repeated sessions in an Asian population, and seems to find a maximal effect in the in the 150–199.9 J range. In our retrospective study we were not able to reproduce such maximal effect with the investigated cumulative energy thresholds, as our results showed increasing efficacy with dose. All but one eye in the high energy group exhibited IOP decrease (Fig. 3). We were able to show that repeated procedures did indeed provide additional benefit without substantial increase in risk (Fig. 1C).

**Table 6:** Overview of reports with calculated cumulative energy values

Article	*n*	Energy/treatment (J)	Cumulative energy (J)
Al Habash et al. (2019) [41]	71	165.26	165.26–330.52
Aquino et al. (2015) [13]	24	62.6	62.6–187.8
Benhatchi et al. (2019) [37]	44	50	100
Chamard et al. (2021) [44]	94	75.1	150.2
Chen et al. (2022) [38]	60	100.16	200.32
Chow et al. (2021) [28]	20	80–100.16	160–200.32
de Crom et al. (2020) [33]	141	100–112.68	205.16–354.93
de Vries et al. (2022) [18]	96	100.16–112.68	200.32–338.04
ELGwaily et al. (2020) [43]	61	125.2–175.3	269.9–346.6
Emanuel et al. (2017) [22]	84	90.144–225.36	90.144–225.36
Issiaka et al. (2022) [46]	39	180	180
Keilani et al. (2020) [39]	20	50	100–125.2
Kuchar et al. (2016) [35]	19	62.6–150.24	125.2–300.48
Laurelle et al. (2021) [34]	55	100.16	100.16
Lee et al. (2017) [47]	27	200.32	200.32
Lim et al. (2021) [26]	43	31.3–112.7	< 150 - ≥200
Logioco et al. (2020) [48]	143	87.64–112.68	175.28–225.36
Magacho et al. (2020) [21]	89	200	200–800
Preda et al. (2020) [40]	100	50.08–81.38	50.08–244.14
Sanchez et al. (2018) [25]	22	62.6–112.68	62.6–112.68
Saraffpour et al. (2019) [16]	73	78.25–62.6	156.5–125.2 (excluded)
Souissi et al. (2021) [45]	37	100	200
Tan et al. (2010) [42]	40	62.6	49.2–146
Tekeli et al. (2021) [30]	76	100–150	200–400
Tekeli et al. (2021) [19]	96	100	200
Varikuti et al. (2019) [49]	61	100	100
Vig et al. (2020) [20]	29	50.08–56.31	56.31–112.68
Williams et al. (2018) [23]	79	75.12–225.36	75.12–225.36
Yelenskiy et al. (2018) [29]	197	112.68–150.24	225.36–300.48
Zaarour et al. (2019) [36]	75	112.68	112.68–225.36
Zbiba et al. (2022) [17]	33	100.16	100.16
This study	82	50.08–200.32	60–400.64

The guidelines [24, 27] provide a useful collection of evidence-based consensus mainly on the MicroPulse (IRIDEX) machine. On the other hand, data regarding the safety and the efficacy of the Supra 810 and Vitra 810 (Quantel Medical) machines are scarce [37, 39, 50, 51]. Our results suggest that similar outcomes can be achieved with the Quantel machines, also broadening treatment options.

Among the aforementioned confounders, the greatest limitation of this study is the high percentage of dropout and data loss during the 12-month follow-up period. This is largely attributable to the study being conducted during the COVID pandemic, and to the consequent lockdown and transfer of medical personnel to COVID care facilities.

## Conclusions

Our findings suggest that repeated SL-TSCPC procedures provide additional IOP lowering effect in the Caucasian population, similar to the Asian cohort described by Lim. Eyes with silicone oil seemed to respond to a greater extent over the period of this study. This study supports the inclusion of cumulative energy scales alongside treatment energy and fluence to better address the safety and the effectiveness of repeated procedures in a standardized fashion.

## Data Availability

The datasets used and/or analysed during the current study are available from the corresponding author on reasonable request.

## Abbreviations

CRVO: Central retinal vein occlusion
CW-TSCPC: Continuous wave transscleral cyclophotocoagulation
IOP: Intraocular pressure
LP: Light perception
NLP: No light perception
POAG: Primary open-angle glaucoma
SD: Standard deviation
SL-TSCPC: Subliminal transscleral cyclophotocoagulation
SLT: Selective laser trabeculoplasty
VA: Visual acuity
